# Improving the Biocompatibility and Functionality of Neural Interface Devices with Silica Nanoparticles

**DOI:** 10.1021/acs.accounts.4c00160

**Published:** 2024-05-30

**Authors:** Delin Shi, Sharada Narayanan, Kevin Woeppel, Xinyan Tracy Cui

**Affiliations:** † University of Pittsburgh, Department of Bioengineering, 4200 Fifth Avenue, Pittsburgh, Pennsylvania 15260, United States; ‡ Center for the Neural Basis of Cognition, 4400 Fifth Avenue, Suite 115, Pittsburgh, Pennsylvania 15213, United States

## Abstract

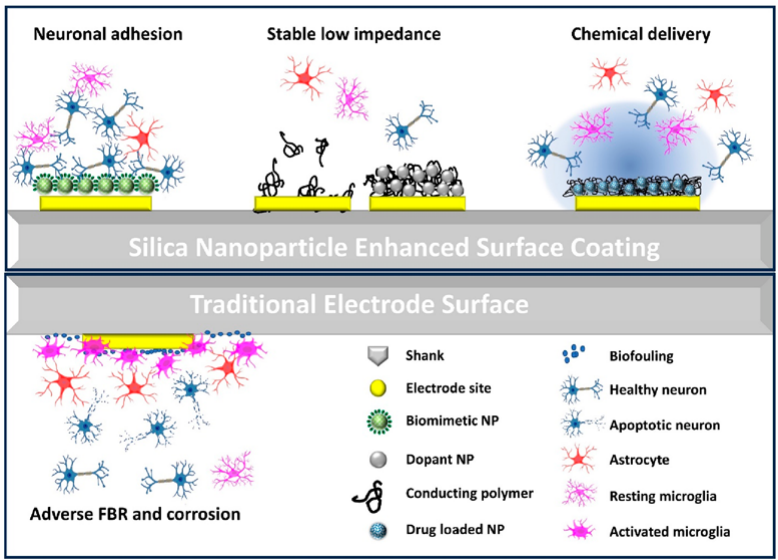

Neural interface technologies enable bidirectional communication between the nervous system and external instrumentation. Advancements in neural interface devices not only open new frontiers for neuroscience research, but also hold great promise for clinical diagnosis, therapy, and rehabilitation for various neurological disorders. However, the performance of current neural electrode devices, often termed neural probes, is far from satisfactory. Glial scarring, neuronal degeneration, and electrode degradation eventually cause the devices to lose their connection with the brain. To improve the chronic performance of neural probes, efforts need to be made on two fronts: enhancing the physiochemical properties of the electrode materials and mitigating the undesired host tissue response.

In this Account, we discuss our efforts in developing silica-nanoparticle-based (SiNP) coatings aimed at enhancing neural probe electrochemical properties and promoting device–tissue integration. Our work focuses on three approaches:

(1) SiNPs’ surface texturization to enhance biomimetic protein coatings for promoting neural integration. Through covalent immobilization, SiNP introduces biologically relevant nanotopography to neural probe surfaces, enhancing neuronal cell attachments and inhibiting microglia. The SiNP base coating further increases the binding density and stability of bioactive molecules such as L1CAM and facilitates the widespread dissemination of biomimetic coatings. (2) Doping SiNPs into conductive polymer electrode coatings improves the electrochemical properties and stability. As neural interface devices are moving to subcellular sizes to escape the immune response and high electrode site density to increase spatial resolution, the electrode sites need to be very small. The smaller electrode size comes at the cost of a high electrode impedance, elevated thermal noise, and insufficient charge injection capacity. Electrochemically deposited conductive polymer films reduce electrode impedance but do not endure prolonged electrical cycling. When incorporated into conductive polymer coatings as a dopant, the SiNP provides structural support for the polymer thin films, significantly increasing their stability and durability. Low interfacial impedance maintained by the conducting polymer/SiNP composite is critical for extended electrode longevity and effective charge injection in chronic neural stimulation applications. (3) Porous nanoparticles are used as drug carriers in conductive polymer coatings for local drug/neurochemical delivery. When triggered by external electrical stimuli, drug molecules and neurochemicals can be released in a controlled manner. Such precise focal manipulation of cellular and vascular behavior enables us to probe brain circuitry and develop therapeutic applications.

We foresee tremendous opportunities for further advancing the functionality of SiNP coatings by incorporating new nanoscale components and integrating the coating with other design strategies. With an enriched nanoscale toolbox and optimized design strategies, we can create customizable multifunctional and multimodal neural interfaces that can operate at multiple spatial levels and seamlessly integrate with the host tissue for extended applications.

## Key References

- WoeppelK. M.; CuiX. T.Nanoparticle and Biomolecule Surface Modification Synergistically Increases Neural Electrode Recording Yield and Minimizes Inflammatory Host Response. Adv. Healthcare Mater.2021, 10( (16), ), 200215010.1002/adhm.202002150PMC837379334190425.^1^*When coupled with L1 neuroadhesive protein, the silica nanoparticle coating can enhance the binding capacity and unique bioactivity of the protein to synergistically improve the chronic neural electrode performance.*
- WoeppelK.; DhawanV.; ShiD.; CuiX. T.Nanotopography-Enhanced Biomimetic Coating Maintains Bioactivity after Weeks of Dry Storage and Improves Chronic Neural Recording. Biomaterials2023, 302, 12232610.1016/j.biomaterials.2023.12232637716282 PMC10993103.^2^*Silica nanoparticles protect the bioactivity of surface-bound L1 protein after storage under dry conditions. This can enable widespread dissemination of L1 and other active biomolecule-coated neural probes.*
- WoeppelK. M.; ZhengX. S.; SchulteZ. M.; RosiN. L.; CuiX. T.Nanoparticle Doped PEDOT for Enhanced Electrode Coatings and Drug Delivery. Adv. Healthcare Mater.2019, 8( (21), ), 190062210.1002/adhm.201900622PMC684206231583857.^3^*Silica nanoparticles as a dopant can significantly enhance the durability and longevity of conductive polymer thin films against prolonged electrical stimulation. Mesoporous nanoparticle dopants enable drug loading and electrically triggered release.*
- WoeppelK. M.; KraheD. D.; RobbinsE. M.; VazquezA. L.; CuiX. T.Electrically Controlled Vasodilator Delivery from PEDOT/Silica Nanoparticle Modulates Vessel Diameter in Mouse Brain. Advanced Healthcare Materials n/a (n/a)2024, 13, 230122110.1002/adhm.202301221PMC1084290837916912.^4^*This in vivo experiment using real-time two-photon microscopy imaging demonstrated that vasodilators loaded into the porous nanoparticle can be electrically released to alter the local vascular dynamics on demand.*

## Introduction

Neural interface devices are placed in the nervous system to facilitate bidirectional communication between the nervous system and external instruments. They enable the recording of physiological signals arising from neurons as well as the delivery of external stimuli to modulate cellular behavior. With advanced neural interfaces, we can systematically monitor activities from multiple neurons at single-cell resolution, map neural circuits, perturb or modulate cellular behavior, and eventually unravel the mechanisms governing high-level brain functions. Such advancements not only open new frontiers for neuroscience research but also hold great promise for novel diagnostic, therapeutic, and rehabilitation approaches for various neurological and psychiatric disorders including stroke, Parkinson’s disease, depression, and spinal cord injury.

Despite the significant advances in neural interface device development, the functional coupling between even state-of-the-art neural probes and neural tissue remains unsatisfactory and especially lacks chronic stability. Beyond the initial traumatic damage inflicted by implantation, the continuous presence of the foreign implant induces a mechanical disturbance and triggers a cascade of inflammatory biochemical events that disrupt the delicate homeostasis of the tissue environment. Such adverse events damage nearby neurons, activate immune cells, and impede device–tissue integration. Additionally, the dynamic and inflammatory tissue environment imposes mechanical and oxidative stress on neural probe materials, causing device degradation and ultimate failure.

**Figure 1:**
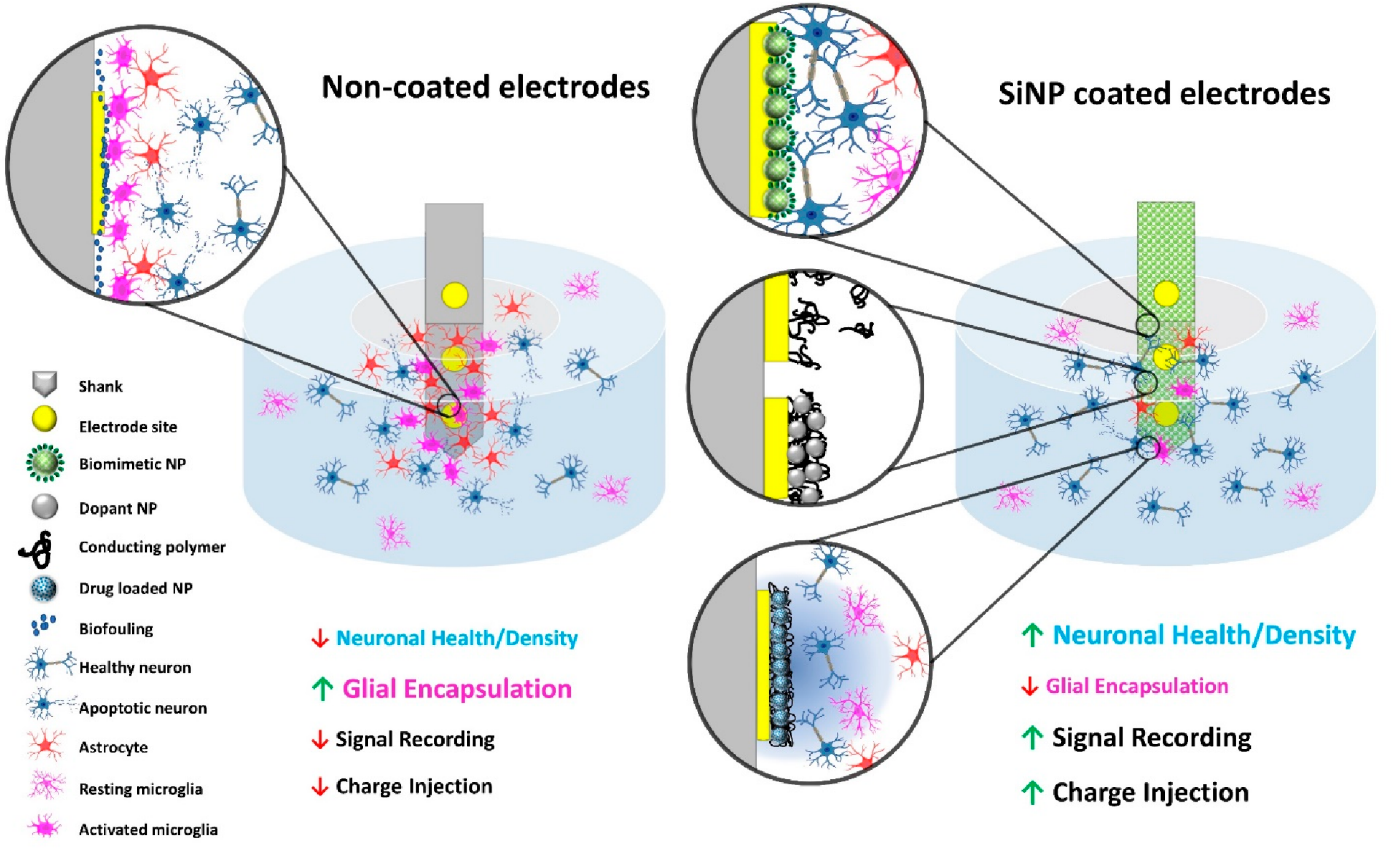
Approaches to utilizing SiNP for enhancing neural probes’ electrochemical properties and promoting device–tissue integration. (Left) The performance of traditional neural probes without any coating is usually unsatisfactory because of their limited electrochemical properties and adverse tissue reactions. (Right) SiNP of different functionalities. (Top to bottom inset) Biomimetic molecules coupled with SiNP can promote neurointegration. When doped into the conductive polymer thin film, the SiNP can provide structural support and protect the polymer from fragmentation and delamination. Mesoporous SiNP can carry drugs/neurochemicals for local electrically controlled chemical delivery and cell manipulation. This figure uses icons from Biorender.com.

**Figure 2:**
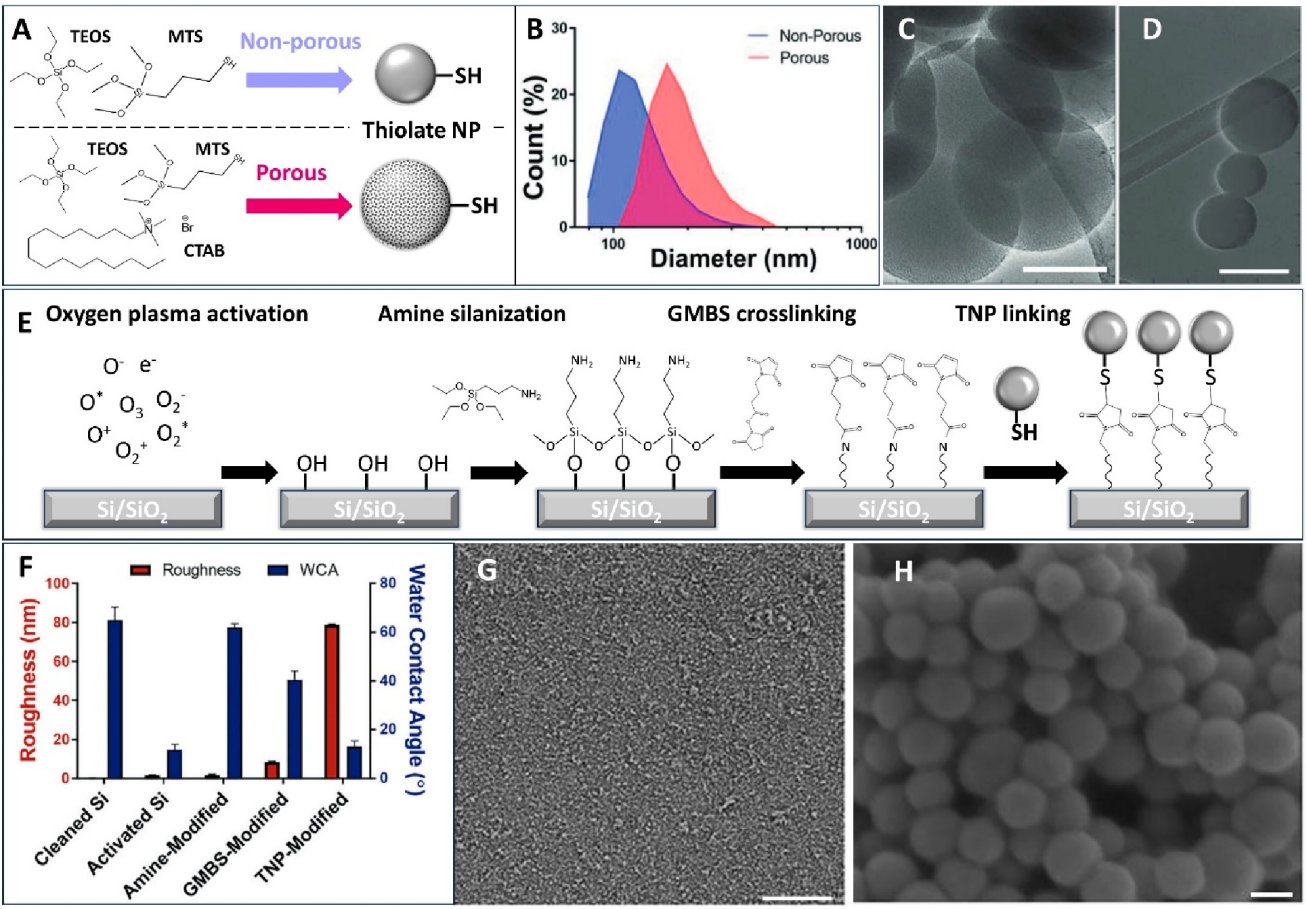
Synthesis, characterization, and immobilization of silica nanoparticles. (A) Synthesis chemistry of nonporous and porous SiNP with thiol functional groups on the surface. (B) Dynamic light scattering measured particle diameter distribution. Transmission electron microscopy imaging of (C) porous particles and (D) nonporous particles. Adapted with permission from ref (3). Copyright 2019 Wiley. (E) Chemistry route to immobilize thiolated nanoparticles to silicon/silicon dioxide substrate. The aminosilane (APTES) reacts with the hydroxyl groups introduced by the oxygen plasma to form a strong silanol bond with the surface. TNP can be covalently linked to the amines via the GMBS linker. (F) Step-by-step surface characterization (left *y* axis, roughness measured by ellipsometry; right *y* axis, water contact angle) during the particle immobilization procedure. Adapted with permission from ref (1). Copyright 2021 Wiley. (G, H) Scanning electron microscopy (SEM) imaging of nonporous particles immobilized on a silicon substrate. Adapted with permission from ref (11). Copyright 2018 Royal Society of Chemistry. Scale bar: (C, D) 100 nm, (G) 100 μm, and (H) 50 nm.

To address these challenges, we developed various silica nanoparticle-based coatings aimed at enhancing neural electrode electrochemical properties and promoting device–tissue integration (Figure 1). Commonly synthesized from silica precursors such as tetraethyl orthosilicate (TEOS), silica nanoparticles (SiNP) possess unique mechanical and chemical properties that can be beneficial for interfacing with brain tissue: (1) silica is biologically inert and biocompatible, (2) the SiNP surface can be easily functionalized through silane chemistry to incorporate bioactive molecules, (3) the size and shape of SiNPs are highly controllable by manipulating the reaction parameters, allowing for optimized interaction between the surface topography and biological components at different length scales, and (4) the particles can be made porous with large specific surface areas and pore volumes, making them highly effective drug carriers (Figure 2A).

Here we discuss our work in SiNP-based neural microelectrode surface coatings in three areas: 1) enhancing the bioactive properties of biomolecule coatings with nanoparticle surface modification, 2) improving the electrode electrochemical properties by incorporating silica nanoparticles as dopants in conductive polymer coatings, and 3) developing an electrically controllable drug-release paradigm with porous silica nanoparticles as drug carriers in conductive polymer coatings (Figure 1). These developments are aimed at enhancing device integration into the host tissue and improving device stability and functionality. Vast engineering possibilities exist for SiNPs and the work presented here is only the beginning of this exploration. In the future, we will continue to utilize SiNPs for the improvement of neural interface technology by incorporating other biological and nanocomponents, new synthesis and functionalization chemistries, and advanced manufacturing technologies.

## Surface Modification with SiNP

The intimate contact between the surface of an implanted electrode device and neural tissue makes the device surface chemistry and topography crucial for good device–tissue integration.^5^ In our earlier work, we developed a biomimetic coating using neuronal cell adhesion molecule L1 (L1CAM) extracted from the neuronal cell membrane.^6^ Covalent immobilization of L1CAM on the neural electrode surface promoted neuronal attachment and survival in the vicinity of the implant and concurrently reduced microglia activation and astrogliosis.^7−10^ Our extended *in vivo* electrophysiological study on mice demonstrated that the L1CAM coating substantially improved the single-unit yield and maintained this improvement for up to 16 weeks.^10^

**Figure 3:**
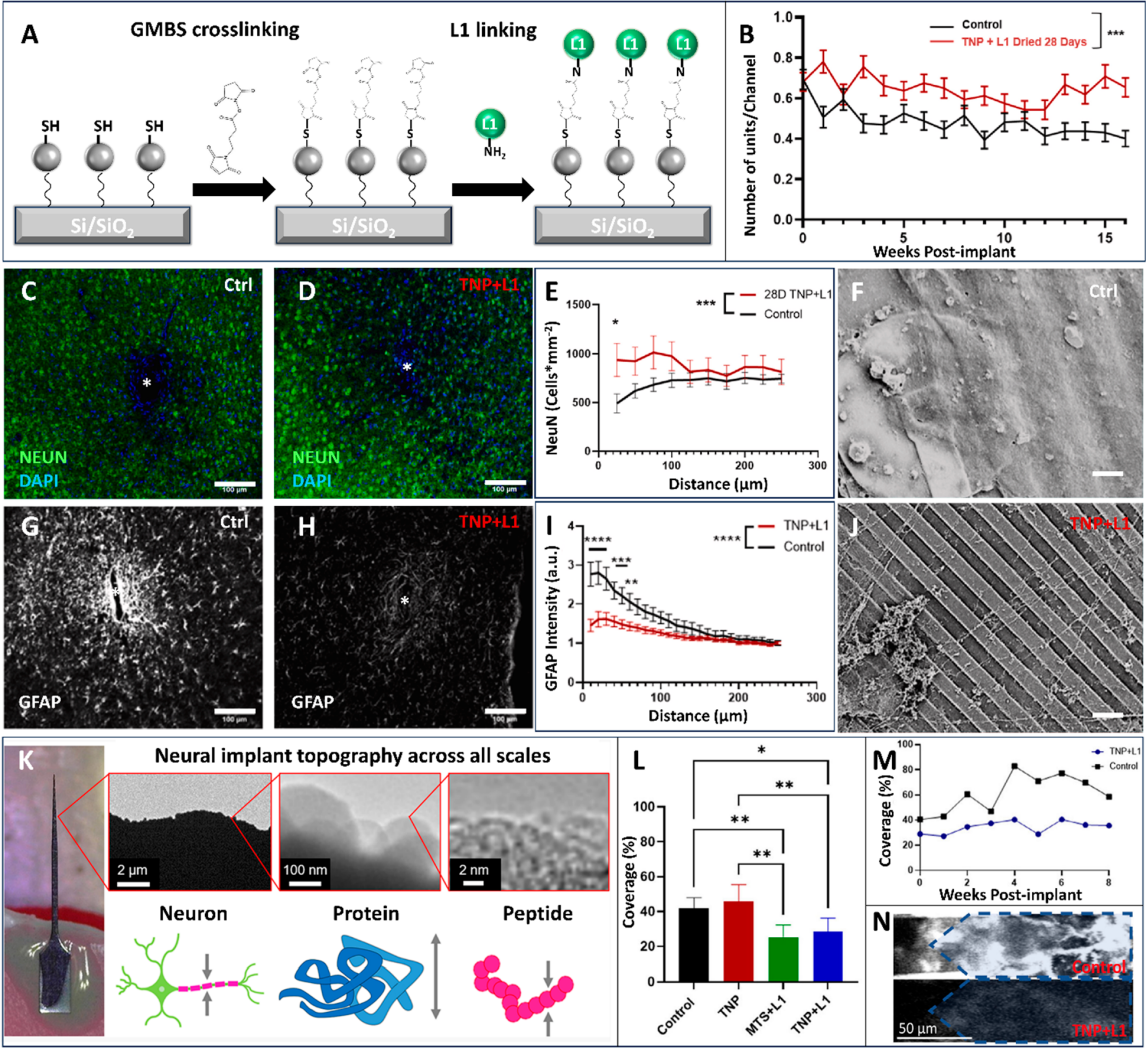
Silica nanoparticles work synergistically with L1 cell adhesion molecules to improve tissue integration. (A) Chemical route using the GMBS linker to covalently bind L1CAM to TNP already immobilized on the substrate. (B–J) Dry-aged TNP+L1 can still improve chronic electrophysiology performance and tissue integration. Adapted with permission from ref (2). Copyright 2023 Elsevier. (B) The number of average single units recorded per channel and mixed model for repeated measurements with Sidak’s multiple comparison tests (*p* < 0.001). (C–E) Representative histology and quantification of NeuN marked neuronal density. (G–I) Representative histology and quantification of GFAP-marked astrocytes. (E, I) Two-way ANOVA with Tukey’s post hoc. (F, J) SEM of attached tissue from explanted uncoated control and TNP+L1-coated probes. (K) Multiscale topography analysis revealed that the roughness increase is biologically relevant, especially for proteins and peptides. Adapted with permission from ref (13). Copyright 2022 American Chemical Society. (L–N) Two-photon imaging reveals decreased microglial coverage of neural electrodes following L1 immobilization. Adapted with permission from ref (12). Copyright 2022 Elsevier. (L) Coverage within 6 h after implanting. (M) Coverage monitored over 8 weeks. (N) Images of coverage after 8 weeks. Scale bars: (C, D, G, H) 100 μm, (F, J) 5 μm, (K) 2 μm to 2 nm, and (N) 50 μm. *p < 0.05, **p < 0.01, ***p < 0.001, and ****p < 0.0001.

While biomolecule immobilization-based coatings show clear benefits, they can be unstable, as proteins and peptides are prone to denaturing and losing their bioactivity. Moreover, their efficacy is limited by the surface area of the smooth electrode substrates. We have developed a method for immobilizing thiol-functionalized SiNP (TNP) onto silicon-based neural probes through silanization and GMBS (*N*-γ-maleimidobutyryl-oxysuccinimide ester) cross-linking chemistry to create a highly texturized surface (Figure 2E). L1CAM is then immobilized onto the TNP-modified surface using GMBS (Figure 3A). Compared to smooth substrates with matching surface chemistry, the TNP-coated substrates demonstrated not only a substantial increase in surface roughness and area (Figure 2F–H) but also a 2-fold increase in protein binding. This heightened surface-bound protein density significantly amplified the beneficial effects of L1CAM as evidenced by increased neurite extension in *in vitro* neuron culture.^11^ Furthermore, we also observed increased neuronal density and reduced microglia activation adjacent to the coated electrodes *in vivo* as well as an elevated single-unit recording channel yield.^1^ In a mechanistic study focusing on how L1CAM modulates microglia activation, we discovered that the TNP+L1CAM coating significantly reduced pro-inflammatory cytokine release, superoxide production, and microglia coverage (Figure 3L–N).^12^

The increased roughness and unique texture of the nanoparticle coating also directly impact the cellular interactions with the implant. To isolate the effect caused by the texture/roughness alone, we compared the growth of neurons and astrocytes on TNP-coated surfaces and thiol-modified, chemistry-matching, smooth surfaces *in vitro*. We observed that increased roughness alone significantly promoted neurite outgrowth^1^ without significantly affecting astroglia coverage.^11^ We also observed that the TNP surface inherently reduced the attachment of large-soma microglia despite no difference in the concentration of inflammatory signaling molecules such as nitric oxide, superoxide, and cytokines.^12^

Moreover, SiNP played a crucial role in stabilizing the immobilized L1CAM protein.^1^ We evaluated the quantity of L1 bound to the substrate by simulating physiological aging conditions (PBS incubation at 37 °C), digesting off the protein that remained bound to the surface, and measuring the protein concentration with UV absorbance. We found that the SiNP-coated substrate not only had more surface-bound protein before aging but also significantly reduced protein loss after aging compared with the smooth surface. Furthermore, as evidenced by *in vitro* neural cell culture, the bioactivity of L1CAM was preserved significantly better on SiNP than on the smooth surface after aging for 28 days.

To comprehensively understand how nanotopography influences biological interactions at different length scales, we conducted multiscale topographic analysis using scanning and transmission electron microscopy. This analysis revealed that the SiNP roughness is beneficial for both cellular attachment and protein anchoring.^13^ At intermediate length scales (μm–nm), SiNP-coated surfaces had significantly increased curvature and true surface area, while at very large (mm) and very small (0.1 nm) length scales the perceived roughness was similar to that of uncoated surfaces. The intermediate length scale where the topographical effects are notable is also the relevant length scale for large biomolecules such as proteins and peptides (Figure 3K). As we further examined how topography promoted neurite outgrowth, we found that the disparity between the increase in true and normalized neurite outgrowth cannot be solely explained by the increase in the amount of protein binding. The roughened texture (increased roughness and curvature) may provide extra attachment points for membrane proteins and cellular structures, promoting more natural cellular integration.

Finally, the nanoscale curvature offers multiple anchoring points per immobilized protein molecule, which may help maintain the native 3D conformation of the protein under harsh conditions. The widespread adoption of our L1CAM coating strategy on electrode surfaces has been hindered by the fragility of the protein. The bioactivity of L1CAM immobilized on smooth surfaces greatly decreases within 3 days of storage under dry conditions, making the delivery and storage of coated devices a practical challenge. We have found that with the NP base layer the L1 coating maintains its bioactivity for up to 8 weeks of dry storage at room temperature.^2^*In vivo* electrophysiological and histological investigation on TNP+L1CAM-coated probes (stored dry for 3 days or 4 weeks) confirmed that the bioactive coating maintained its functionality in three key areas: 1) improving chronic recording performance (Figure 3B), 2) promoting neuronal health (Figure 3C–F,J), and 3) reducing gliosis (Figure 3G–I). Through nanoparticle surface modification, we have established a feasible method to commercialize and disseminate biomimetically coated neural interface devices worldwide.

## Enhancing Conductive Polymer Properties as Dopants

As neural interface devices decrease in size and increase in site density, the electrode impedance unfortunately increases. High impedance leads to elevated noise in electrophysiological recording and reduced charge injection capacity. Conductive polymers such as PEDOT and polypyrrole have been used as coatings for microelectrodes to reduce electrode impedance and increase charge injection limit. This is due to their high ionic and electrical conductivity and very high effective surface area.^14^ Conductive polymer coatings are often electrochemically polymerized and deposited onto electrodes in the presence of a negatively charged dopant. The dopant balances out the positive charges along the backbone of the conducting polymer. By the choice of different dopant molecules, conductive polymer coatings can be imparted with unique properties. For example, large bioactive molecules and even cells can be doped into the polymer matrix to permanently impart bioactivity to the device surface, while smaller dopant molecules can be electrochemically loaded and released for drug-delivery applications.^15−17^

**Figure 4:**
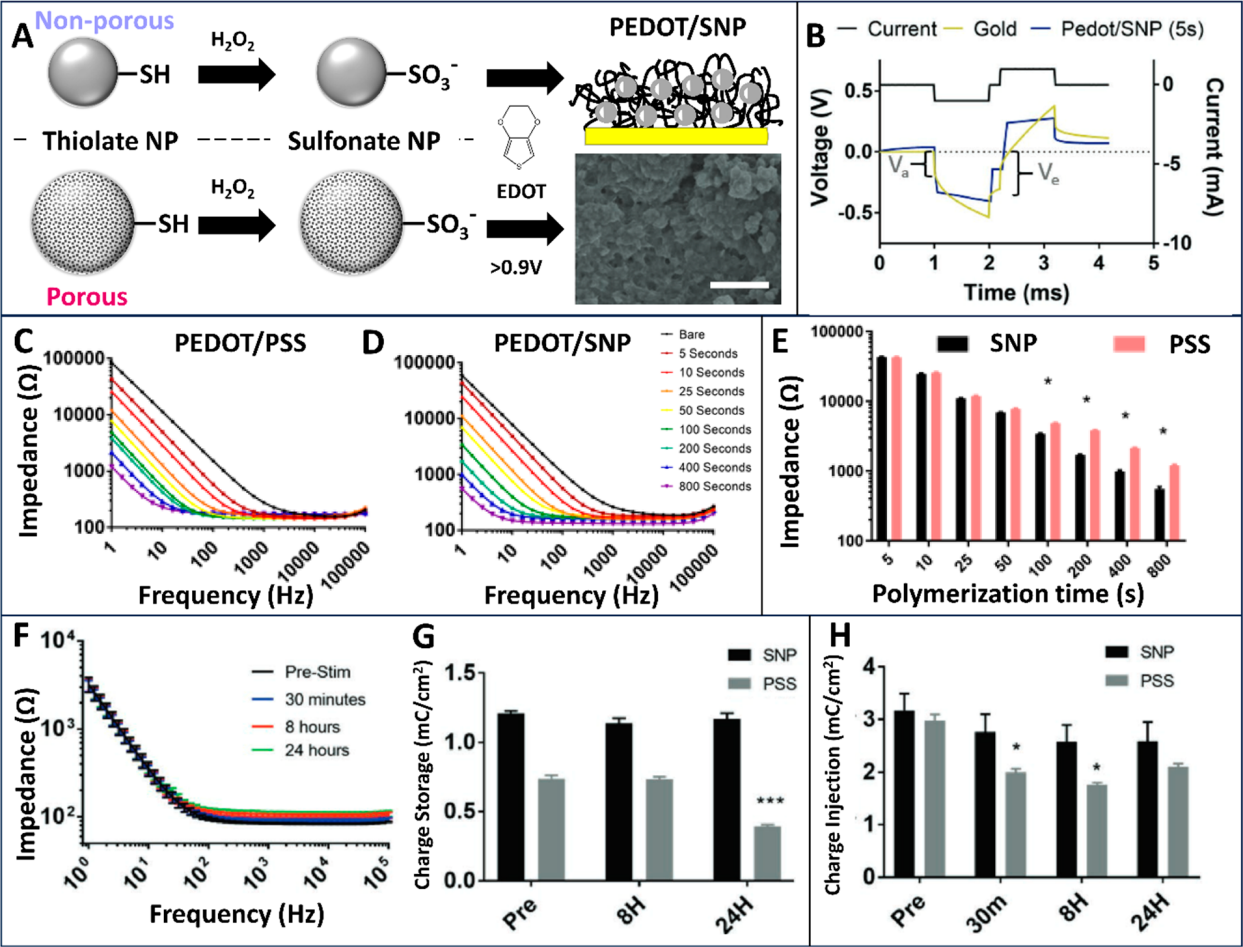
Silica nanoparticles as dopants can improve the stability and durability of the conductive polymer thin film. (A) Thiol groups on the nanoparticle (both nonporous and porous) can be converted to sulfonate groups by oxidation. Then sulfonated nanoparticles can work as dopants to form the PEDOT/SNP composite. The SEM inset is PEDOT/porous SNP. Scale bar 1 μm. (B) Stimulation waveform for charge injection and corresponding voltage transients collected from bare gold or PEDOT/SNP sites. Va is the access voltage and Ve is the electrode voltage for the bare gold electrode and is used to determine the charge injection limit. (C–E) EIS for PEDOT/PSS and PEDOT/SNP polymerized for different times on 2 mm gold electrodes at 10 μA. With longer polymerization times, PEDOT/SNP produces substantially lower impedances, especially in the lower frequency range. (E) 1 Hz impedance of 556 Ω for PEDOT/SNP vs 1203 Ω for PEDOT/PSS after 800 s. Electrochemical characterization: (F) EIS, (G) charge storage capacity, and (H) charge injection limit for PEDOT/SNP (nonporous) films before and after stimulation for 30 min, 8 h, and 24 h at 50 Hz. (A–H) Adapted with permission from ref (3). Copyright 2019 Wiley. *p < 0.05, **p < 0.01, ***p < 0.001, and ****p < 0.0001. Significance in (G, H) is relative to prestim samples.

The most commonly used dopant for PEDOT is polystyrenesulfonate (PSS). While PSS offers PEDOT high conductivity, the electrochemical stability of the PEDOT/PSS coating remains a significant challenge, especially in chronic stimulation applications. By functionalizing SiNP with sulfonate groups, we created highly negatively charged SiNPs suitable for doping into PEDOT (Figure 4A). The most notable advantages of using SiNPs as dopants include improved stability of the electrode coating and the ability to controllably release a wide spectrum of drugs (as discussed in detail in the next section). We examined the electrochemical properties of PEDOT doped with sulfonate-SiNPs (SNPs). Long-term stimulation at high current amplitude had no detrimental effect on the charge storage capacity (CSC) and current injection limit (CIL) of the PEDOT/SNP films. This is a significant improvement over the classical PEDOT/PSS electrode coatings which show a major drop in CSC and CIL after chronic stimulation (Figure 4G,H).^18^ Moreover, the starting CIL of the PEDOT/SNP-coated probes is much higher than that of the PEDOT/PSS-coated electrodes (Figure 4G,H). SEM imaging of the PEDOT/SNP films after 300 CV scans in a PBS buffer showed no structural damage to the film or delamination. We attribute the remarkable stability of the PEDOT/SNP films to the large size and rigid structure of the nanoparticles, reducing the mechanical impact of repeated film swelling and shrinkage during stimulation, as well as to the high van der Waals interactions between the charged SiNP and the substrate.^3^

## Controlled Drug Release

**Figure 5:**
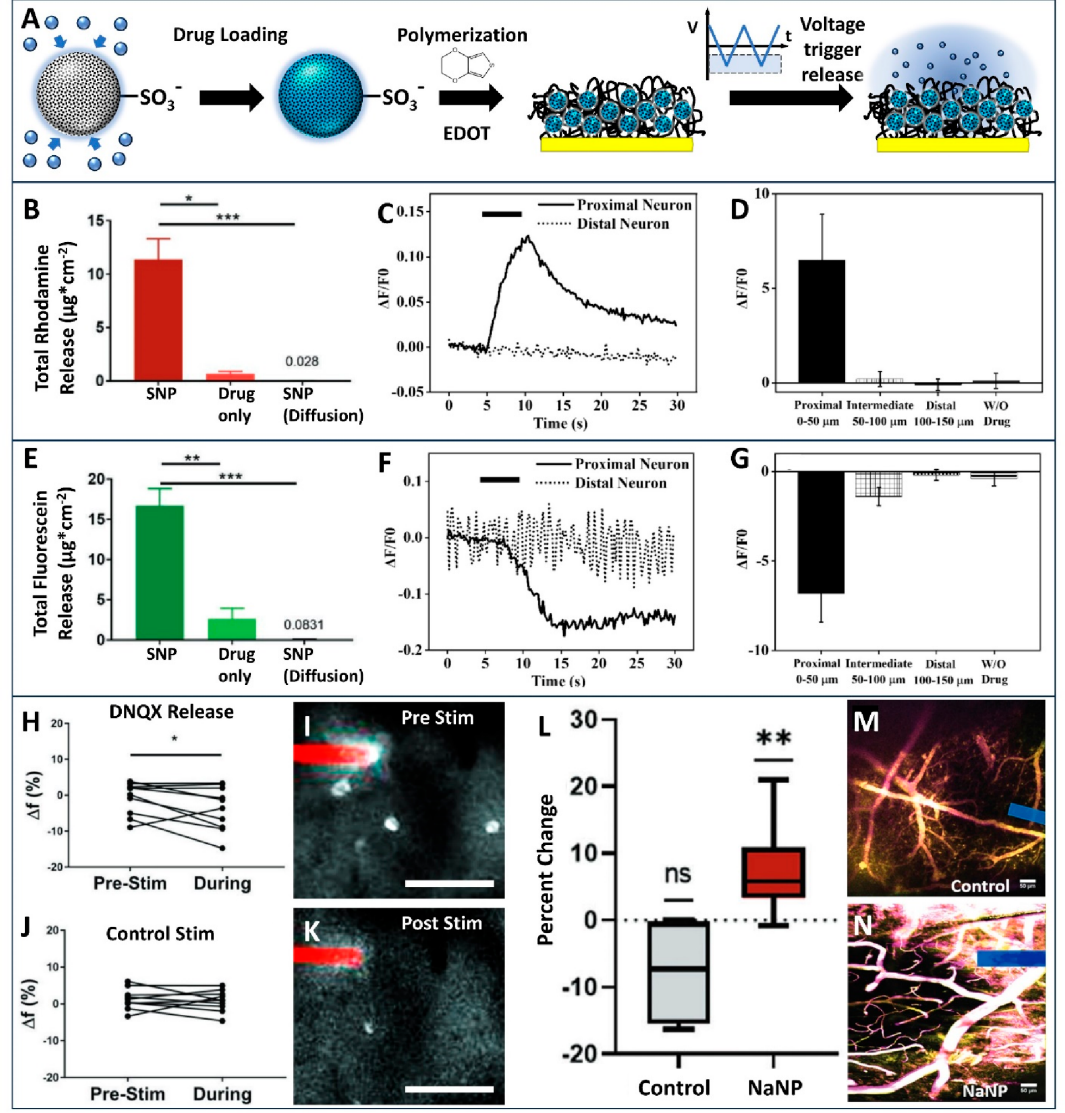
Silica nanoparticles used as cargo carriers for the controlled delivery of drugs and neurochemicals. (A) Drug loading and release. Drugs are loaded into porous nanoparticles via sonication. Loaded particles are polymerized with EDOT to produce a composite thin film. The release can be triggered with a triangular cyclic voltage sweep with sufficient reducing voltage. (B, E) Comparison of total drug release of fluorescein and rhodamine from active release from PEDOT/SNP, active release of the drug without SNP, and passive diffusion from PEDOT/SNP. Adapted with permission from ref (3). Copyright 2019 Wiley. (C, D, F, G) Fluorescent calcium imaging of neuron activities upon electrically triggered neurochemical release from a PEDOT/SNP-coated microwire carrying (C, D) GLU or (F, G) GABA. Adapted with permission from ref (26). Copyright 2021 by the authors. (H–K) Quantification of GCaMP activity before and during stimulation through (H) a DNQX-loaded electrode or (J) an unloaded electrode. (I, K) Pixel standard deviations for prestimulation and during-stimulation frames for a DNQX-loaded electrode, respectively. Image contrast is enhanced for easier visualization. Adapted with permission from ref (3). Copyright 2019 Wiley. (L–N) Two-photon imaging of the effect of electrically released vasodilator NaNP. (L) Percent diameter change of large vessels (diameters greater than 65 μm, *n* = 6 for control and *n* = 9 for NaNP). (M, N) Overlays of cortical vessels before (yellow) and after (magenta) stimulation for the control and NaNP group. Adapted with permission from ref (4). Copyright 2023 Wiley. Scale bars: (I, K) 100 μm and (M, N) 50 μm. *p < 0.05, **p < 0.01, ***p < 0.001, and ****p < 0.0001.

Adding a surfactant to the SiNP synthesis solution produces mesoporous SiNPs (mSiNPs), which have been shown to be excellent drug carriers in numerous studies.^19−21^ By functionalizing mSiNPs with sulfonate groups, we were able to dope mSiNPs into PEDOT thin film coatings and develop a versatile electrically controlled drug release paradigm. Due to the electroactivities of the PEDOT and the electrostatic nature of the PEDOT/dopant interaction, switching the potential of the electrode between positive and negative can cause changes to the redox states of the conducting polymer as well as the affinity of the dopant for the polymer. These changes often trigger ionic and water movement in and out of the polymer film and consequent volumetric changes (Figure 5A).

The first generation of electrically triggered drug release was performed by directly using negatively charged small-molecule drugs as dopants in conducting polymer electrode coatings. The drug release was accomplished by applying a negative current/potential to the electrode. While effective, such a drug release system is limited in the quantity, charge, and type of drug that can be loaded and released. To improve the drug-loading capacity, we have previously loaded drugs into nanoreservoirs such as graphene oxide nanosheets and carbon nanotubes which are then incorporated into conducting polymer coatings during polymerization.^22−25^ While some of these nanocarrier-based drug release systems allow drug molecules of positive and negative charges to be released, the process of electropolymerization may oxidize and degrade electroactive drug molecules. To overcome this limitation, we use negatively charged mesoporous silica nanoparticles as drug reservoir dopants for conducting polymers. The mSiNPs are loaded with drug by sonicating the charged mesoporous NPs in drug solution. The NPs are then suspended in an EDOT solution and electropolymerized. The drug is released from the NP pores during electrical stimulation when the oxidative and reductive potentials cause expansion and contraction of the polymer film due to the influx and outflux of water and ions. Thus, neither drug loading nor release relies on specific charge conditions of the drug molecules. Due to the ability of nonconductive SiNPs to shield drugs from oxidation during polymerization, electroactive drug molecules such as melatonin and sodium nitroprusside can be loaded and released without losing efficacy.^3,4^

We have demonstrated the ability to load and controllably release several drugs of choice from PEDOT/SNP electrode coatings using cyclic, square, and sinusoidal waveforms.^3,4,26,27^ For visualization of the drug release, we showed the loading and release of fluorescein (negatively charged) and rhodamine (positively charged), two oppositely charged and fluorescent compounds, from the PEDOT/SNP films. Both drugs were released successfully on the order of 10 μg/cm^2^ of polymer film per stimulation cycle (Figure 5B,E).^3^ We also loaded neurotransmitters including glutamate and GABA into PEDOT/SNP microwire coatings and demonstrated in vitro neuronal modulation using fluorescent Ca^2+^ imaging in neuronal cell culture (Figure 5C,F). We noted that the neuromodulation effects of the drug delivery extended only to neurons within 50 μm of the microwire, demonstrating the high spatial localization of the drug release (Figure 5D,G).^26^

*In vivo*, we used two-photon microscopy to visualize the effect of DNQX, a glutamate transmission blocker, on the neural activity of a GCaMP mouse. We observed significantly lower neural firing activity after the release of DNQX, demonstrating a direct, measurable effect of the drug release on the surrounding neurons (Figure 5H,J).^3^ Other small-molecule neuromodulating drugs that we have successfully loaded and released using PEDOT/SNP coatings include melatonin, doxorubicin, dopamine, and bicuculline.^3,4,26,27^

We have applied the PEDOT/SNP controlled-drug release technology for the *in vivo* release of sodium nitroprusside (NaNP), a vasodilator, from carbon fiber electrode (CFE) surfaces.^4^ Vascular damage due to electrode implantation is a major source of neurodegeneration around the probe.^28,29^ The delivery of a vasodilator has the potential to prevent neurodegeneration and accelerate neural recovery after probe implantation by increasing the blood flow to the damaged area. After performing *in vitro* tests to confirm the controllable release of NaNP from the PEDOT/SNP-coated probe, we used two-photon imaging to observe the effect of the drug release in a cortically implanted mouse. We used an SR101 injection to visualize blood vessels in the brain and observed a significant increase in the mean blood vessel diameter during drug release in blood vessels larger than 20 μm in diameter (Figure 5M,N). Blood vessels smaller than 20 μm in diameter were not modulated by drug release (Figure 5L). As NaNP acts on smooth muscle cells to promote vessel expansion, these results correlate with the fact that capillaries, the smallest classification of blood vessels, do not contain smooth muscle cells.^4^

We have also incorporated the PEDOT/SNP drug release coating on flexible neural probes to enable simultaneous electrophysiological recording and chemical neural modulation. Flexible probes were fabricated with flexible Parylene-C shanks, 16 PEDOT/PSS-coated recording microelectrodes, and 2 extra-large chemical site electrodes for drug loading and release. The chemical sites were coated with PEDOT/SNP loaded with small-molecule neurotransmitters gamma-aminobutyric acid (GABA – inhibitory) or glutamate (Glu – excitatory). The probes were implanted into layer IV of the barrel cortex of rats. For simultaneous drug delivery and electrophysiological recording, we used a slow sine wave stimulus to minimize the electrical modulation of neurons. We observed a significant decrease in the spike rate after GABA release and a significant increase in the spike rate after Glu release.^27^ The ability to modulate neural activity transiently and repeatedly in a highly localized manner *in vivo* provides a powerful tool for neural circuit analysis and cell-type identification.

## Conclusions and Outlook

The utilization of SiNP-based surface modifications discussed in this Account exemplifies their potential to greatly advance the functionality and performance of neural interfaces. Through surface modification, SiNP introduces biologically relevant nanotopography onto neural implant surfaces, influencing cell–substrate biomechanical interactions. SiNP nanotopography also enhances bioactive surface coatings such as the L1CAM protein coating by increasing the surface binding density, stabilizing protein bioactivity, and synergistically amplifying the efficacy of the coating in promoting seamless implant-tissue integration. The SiNP coating facilitates the widespread dissemination of biomimetic coatings by maintaining protein bioactivity under harsh transport conditions.^1,2,11−13^

By functionalizing SiNP with sulfonate groups, SiNP can become an excellent dopant for conducting polymers. Electrochemically deposited conductive polymer films have demonstrated remarkable effectiveness in reducing electrode site impedance and increasing the charge injection limit, but they cannot endure prolonged electrical cycling. The rigidity of SiNP dopants in PEDOT films provides structural support to the thin polymer film, significantly increasing its chronic stability and durability upon electrical stimulation. The low interfacial impedance maintained by the conducting polymer/SiNP composite is critical for extended electrode longevity and effective charge injection for chronic applications.^3^

Finally, we have demonstrated a versatile electrically controlled drug release system by utilizing mesoporous sulfonated SiNP drug carriers as dopants for conducting polymers. Such precise focal manipulation, in vascular modulation or exciting/inhibiting neuronal activities, not only enables us to probe the brain circuitry and neurovascular coupling but also promises clinically translatable therapeutic applications. Compared to previous electrochemical drug release methods from conducting polymers, drug delivery from PEDOT/SiNP films allows the loading of drugs with a variety of charge states and protection of the payload from electrochemical damage during electropolymerization.

**Figure 6:**
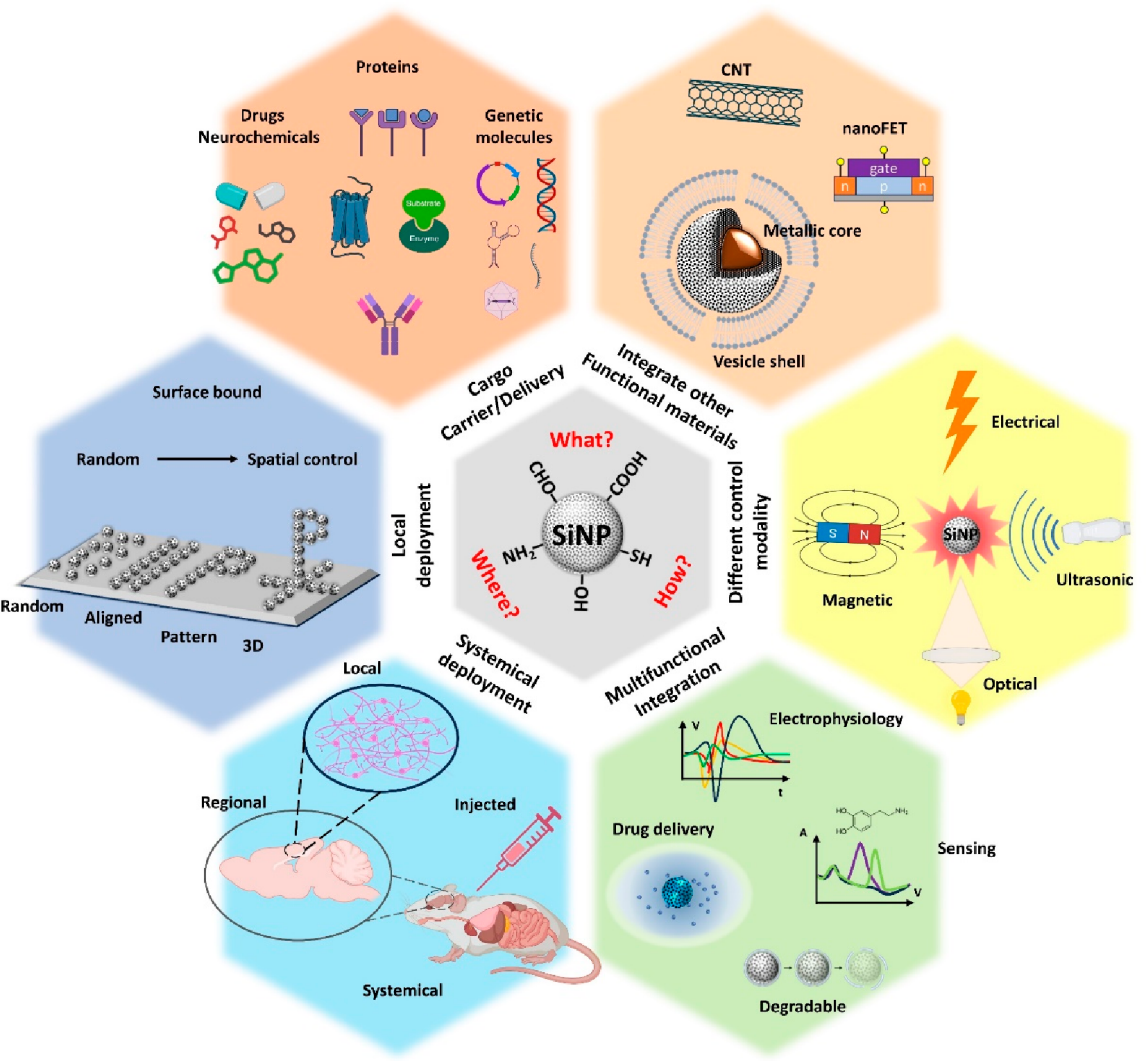
Future directions of SiNP applications. SiNPs can be used to carry or deliver a wide range of biofunctional molecules including drugs, neurochemicals, proteins (receptors, enzymes, antibodies, etc.), and genetic molecules (DNA, RNA, plasmids, aptamers, viruses, etc.). SNPs can also be made more complex (i.e., vesicle shell, metallic core, etc.) and can be integrated with other functional materials such as carbon nanotubes and other electronic parts such as nanoFET. Engineered SiNPs can be activated with versatile remote-control modalities. They can be deployed locally from the surface of neural implants with precise spatial control or injected locally or systemically to interface with regional tissue or the full body. SiNPs will accelerate the development of next-generation neural interfaces that are customizable, multifunctional, and multimodal and can operate at multiple spatial levels for extended time. This figure uses icons from Biorender.com.

The functionalities of SiNP-based nanomaterials explored in our work represent only a small fraction of their tremendous potential. While many excellent reviews on recent advances in nanomaterials for neural interfaces exist,^30−34^ we would like to briefly discuss a few future directions below (Figure 6). The highly tunable size, porosity, and surface chemistry of SiNP make it an ideal platform for carrying or incorporating functional bioelements. Delivering genetic macromolecules through SiNP for permanent transfection or transient expression is highly effective, specific, customizable, and safe.^19,21,35−38^ By leveraging more sophisticated genetic circuit-controlling strategies established in the field of synthetic biology, we could achieve more precise manipulation of cell fate, function, and behavior.^39^ The ease of chemical functionalization of SiNP offers endless possibilities for incorporating biorecognition elements such as enzymes, aptamers, antibodies, and receptors to enable electrochemical sensing of target molecules via neural electrodes, adding another modality for neural interfacing.^40^ The synergy found between SiNPs and L1CAM could extend to these biorecognition molecules, thereby enhancing their bioactivity and stability for enhanced biosensing capability. Other nanobuilding blocks and nanotools that have been used for neural interfaces can also benefit from SiNPs. Nanoscale field-effect transistors (nanoFET), which are more responsive and sensitive to voltage transients than passive electrodes, have been incorporated into neural probes to form nanoscale junctions with neurons and work like “artificial synapses”.^41−43^ Incorporating biofunctional and nanotextured SiNP materials as a biomimetic coating or biorecognition gating material in the design of nanoFETs can help maintain intimate device–tissue integration and augment their biosensing capabilities.

The basic principle of gating drug release from nanoparticle probe coatings can be extended to a variety of different stimuli. For example, photoresponsive chemical linkers (such as those containing quantum dots, azobenzene moieties, or nucleic acids) can be synthesized to block mesoporous SiNP pores and undergo bond cleavage or conformational changes upon light exposure to open the pores.^44−46^ Light delivery technologies adopted from optogenetics such as waveguides on probes or external light sources coupled with optical fibers can be used to trigger the drug release mechanism.^47,48^ Similar chemical capping mechanisms have been developed that block SiNP pores and uncap upon photothermal heating from NIR light.^49^ Temperature-responsive polymers/hydrogels that reduce volume with heat have also been developed for controlled drug release from mesoporous SiNPs.^50−53^ Localized heating can come from sources such as the integration of IR/NIR light with waveguides or external sources, magnetic field stimulation of superparamagnetic nanoparticles incorporated on the electrode shank or within the SiNPs, and heating of similarly incorporated metal nanoparticles using light.^50,54−56^ Ultrasound stimulation can also trigger drug release from polymers and vesicles primarily through mechanical cavitation of bubbles in the polymer and vesicles.^57,58^ These components can be incorporated into SNPs for less-invasive drug release via ultrasound stimulation. All of the above are examples of how the drug-release technology discussed in this Account can be expanded to respond to diverse, customizable stimuli.

Currently our SiNP surface immobilization uniformly coats the entire probe and lacks precise spatial control over coating patterns. In contrast, highly organized 2D patterns such as aligned lines or grooves can provide oriented guidance cues to direct cellular migration and neurite extension.^59^ The incorporation of two-photon polymerization and SiNP composite ink in 3D printing can enable the patterning of nanoarchitectures at sub-200-nm resolution.^60^ This improvement in spatial control can allow for more intricate and strategically controlled biomechanical and biochemical interactions with brain cells.

Lastly, beyond the surface-confined local delivery, drug-loaded SiNP can also be injected into the bloodstream and targeted to the central nervous system for therapeutic purposes.^20,61,62^ SiNP are well known for their biocompatibility and biodegradability and have been widely used for systemic drug-delivery applications. When integrated with the optical, acoustic, or magnetic remote controlling modalities, systemically delivered SiNP not only can deliver chemicals but also can be used for noninvasive neural modulation.^32^

Our work on nanoparticle surface modifications to neural probes is a rich combination of cutting-edge research in neural engineering, materials science, chemistry, and neuroscience. Continued collaborative and innovative research will facilitate the development of multifunctional, multimodal devices that are capable of electrophysiological recording, stimulation, drug delivery, electrochemical sensing, and versatile remote-control modalities. By leveraging the different effective radii of various deployment strategies, ranging from substrate-bound nanomaterials to systemically delivered nanoparticles, we can also achieve functional operation on multiple spatial levels simultaneously. As we expand the nanofunctional material library and improve our understanding of how brain cells interact with nanoscale features and nanomaterials, we can create long-lasting, high-fidelity, multifunctional, and multimodal neural interfaces customized for any application aimed at advancing neuroscience research or bioelectronic medicine.
